# Developmental progression of respiratory dysfunction in a mouse model of Dravet syndrome

**DOI:** 10.1172/jci.insight.184231

**Published:** 2025-09-09

**Authors:** Brenda M. Milla, Eliandra N. da Silva, Cleyton R. Sobrinho, Monica L. Strain, Daniel K. Mulkey

**Affiliations:** Department of Physiology and Neurobiology, University of Connecticut, Storrs, Connecticut, USA.

**Keywords:** Cell biology, Neuroscience, Epilepsy, Ion channels, Respiration

## Abstract

Dravet syndrome (DS) is an early-onset epilepsy caused by loss-of-function mutations in the *SCN1A* gene, which encodes Nav1.1 channels that preferentially regulate activity of inhibitory neurons early in development. DS is associated with a high incidence of sudden unexpected death in epilepsy (SUDEP) by a mechanism that may involve respiratory failure. Evidence also shows that loss of *Scn1a* impaired activity of neurons in the retrotrapezoid nucleus (RTN) that regulate breathing in response to CO_2_/H^+^, suggesting breathing problems precede seizures and serve as a biomarker of SUDEP. Consistent with this, we showed that *Scn1a^+/–^* mice exhibited a blunted ventilatory response to CO_2_/H^+^ prior to overt seizure activity that worsened with disease progression. Later in development, some *Scn1a^+/–^* mice also showed a blunted ventilatory response to hypoxia. Importantly, the severity of respiratory problems correlated with mortality. We also found that pharmacological activation of Nav1.1 rescued activity deficits of RTN neurons in *Scn1a^+/–^* mice. We conclude that disordered breathing may be an early biomarker of SUDEP in DS, and at the cellular level, loss of *Scn1a* disrupts RTN neurons by mechanisms involving disinhibition and pharmacological activation of Nav1.1 to reestablish inhibitory control of RTN neurons rescues activity deficits.

## Abstract 2

Here, we identify a substrate that contributes to disordered breathing in Dravet syndrome and establish breathing problems as an biomarker of mortality in this disease.

## Graphical abstract

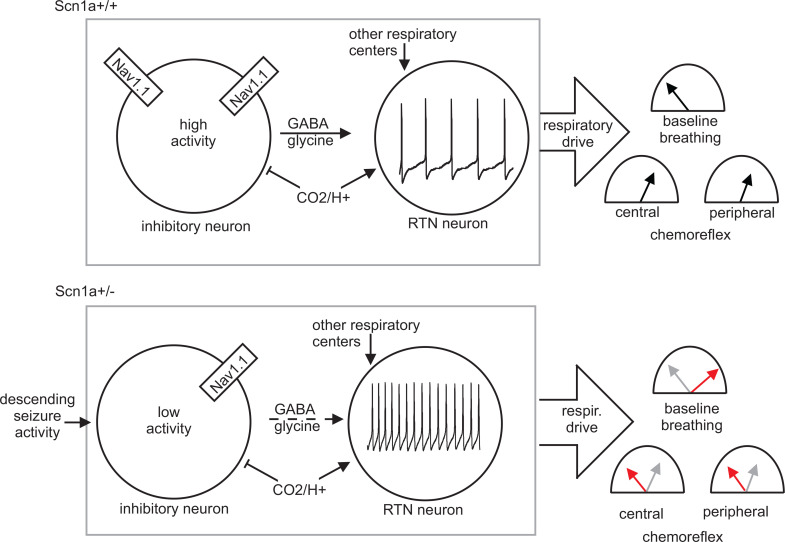

## Introduction

Dravet syndrome (DS) is a severe type of developmental and epileptic encephalopathy characterized by febrile seizures during the first year of life, uncontrolled seizures as toddlers, and a high incidence of childhood sudden unexpected death in epilepsy (SUDEP). Most DS cases (~90%) are caused by loss-of-function mutations in one allele of the *Scn1a* gene (1), resulting in haploinsufficiency of the pore-forming subunit of a voltage-gated Na^+^ channel (Nav1.1) (1). Nav1.1 channels produce a depolarizing current important for initiation and maintenance of neural activity, and since Nav1.1 channels are preferentially expressed by inhibitory neurons early in development (2, 3) and during peak rate of mortality (4), the more severe aspects of DS are thought to result from diminished activity of inhibitory neurons (3, 5). Despite this, current treatments, including those that promote GABAergic signaling, show only modest effectiveness in DS; consequently, DS patient prognosis remains poor and the mean age of SUDEP is just 4.6 years (6). This grim statistic underscores the urgent need for a better understanding of mechanisms contributing to mortality in DS.

Respiratory failure is thought to contribute to SUDEP in DS (7, 8); however, it is not known whether breathing problems occur early in disease onset or can be used as a biomarker of mortality. Therefore, a major objective of this study was to characterize breathing in *Scn1a^+/–^* mice early in development prior to overt seizure activity and at a later time point that coincides with rapid onset of mortality. Also, since patients with DS exhibit a blunted ventilatory response to CO_2_ (i.e., central chemoreflex) (7) and a mouse model of DS identified chemosensitive neurons in the retrotrapezoid nucleus (RTN) as a potential substrate responsible for breathing problems in DS (8), we determined how genetic loss and pharmacological rescue of *Scn1a* function impacts baseline activity and CO_2_/H^+^ sensitivity of RTN neurons.

## Results

### Respiratory phenotype of neonatal Scn1a^+/–^ mouse pups on a mixed background.

**Figure 1:**
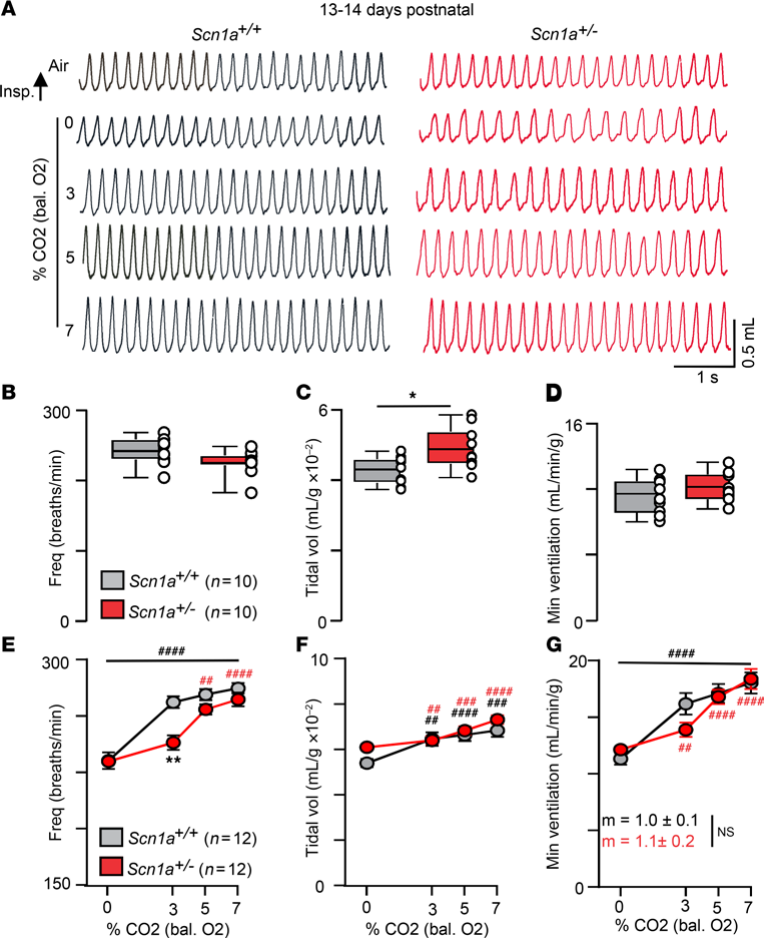
Two-week-old *Scn1a^+/–^* mice show a blunted ventilatory response to moderate hypercapnia (3% CO_2_). (**A**) Traces of respiratory activity from a control and *Scn1a^+/–^* mouse during exposure to room air and graded increases in CO_2_ (balance O_2_). (**B**–**D**) Summary data (*n* = 10/genotype) plotted as mean ± maximum/minimum show small increases in tidal volume (**C**) (*T*_16_ = 2.6) in *Scn1a^+/–^* mice but otherwise normal respiratory frequency (**B**) (*P* = 0.06) and minute ventilation (**D**) (*P* = 0.36) between genotypes under room air conditions (unpaired *t* test). (**E**–**G**) Summary data (*n* = 12/genotype) plotted as mean ± SEM of frequency (**E**) (*P* = 0.0069 for 3% CO_2_ frequency), tidal volume (**F**) (*P* = 1.0), and minute ventilation (**G**) (*P* = 0.20) show that *Scn1a^+/–^* mice fail to increase respiratory frequency in response to a modest increase in CO_2_ (3%) by an amount proportional to control; however, genotype differences were negated at higher CO_2_ levels. Means were compared using repeated measures 2-way ANOVA followed by Tukey’s or Dunnett’s multiple-comparison test and slopes of minute ventilation response to 0%–7% CO_2_ were compared using 1-way ANCOVA. *P < 0.05, **P < 0.01 with Tukey’s multiple-comparison test for differences between genotypes; ^##^*P* < 0.01, ^###^*P* < 0.001, ^####^*P* < 0.0001 with Dunnett’s multiple-comparison test for within-genotype differences from control.

*Scn1a^+/–^* mice (50% C57BL/6J:50% 129/SvJ background) were obtained at the expected frequency and are similar to control animals in terms of weight (*Scn1a^+/+^* 7.1 ± 0.30 g; *Scn1a^+/–^* 7.8 ± 0.2 g; *T*_32_ = 1.9 [where 32 = degrees of freedom], *P* = 0.0589) during the first 2 weeks of life. However, despite their normal appearance, previous work suggests 2-week-old *Scn1a^+/–^* mice are prone to febrile seizures (9), a hallmark feature of DS potentially triggered by heat-induced respiratory alkalosis (10, 11). Prompted by this observation, we characterized baseline breathing and the CO_2_ ventilatory response in 2-week-old mice of each genotype. Note that all in vivo experiments were performed during a time of day associated with SUDEP in people (12), while controlling for state of arousal by assessing breathing only when mice appeared to be in a state of quiet wakefulness. We found that approximately 2-week-old *Scn1a^+/–^* mice show fairly normal respiratory activity under room air conditions (*n* = 10, mixed sex per genotype); baseline parameters of interest include respiratory frequency (*Scn1a^+/+^* 238 ± 6.1 bpm; *Scn1a^+/–^* 221 ± 5.5 bpm) and tidal volume, which was higher in *Scn1a^+/–^* mice (*Scn1a^+/+^* 0.04 ± 0.001 μL/g; *Scn1a^+/–^* 0.05 ± 0.002 μL/g) but otherwise normal minute ventilation (*Scn1a^+/+^* 10.2 ± 0.4 μL/min/g; *Scn1a^+/–^* 10.7 ± 0.4 μL/min/g) (Figure 1, A–D). However, at this same developmental time point, *Scn1a^+/–^* mice showed a diminished capacity to increase respiratory frequency in response to a modest 3% increase in CO_2_ (*Scn1a^+/+^* 257 ± 4.6 bpm vs. *Scn1a^+/–^* 224 ± 7.7 bpm; *F*_1,22_ =5.498, *P* = 0.0069) (Figure 1E). This chemoreceptor deficit normalized at higher levels of CO_2_ (5% and 7% CO_2_) such that respiratory frequency (7% CO_2_ *P* = 0.45), title volume (7% CO_2_ *P* = 0.82), and minute ventilation — the product of respiratory frequency and tidal volume — measured over CO_2_ levels ranging from 0% to 7% (CO_2_ slope *Scn1a^+/+^* 1.0 ± 0.1 vs. *Scn1a^+/–^* 1.1 ± 0.2; *P =* 0.60) were similar between genotypes (Figure 1, E–G). Since *Scn1a^+/–^* mice do not exhibit overt seizures at this developmental time point, it is tempting to speculate that loss of *Scn1a* directly disrupts central chemoreceptor function. However, we also found that seizure-free *Scn1a^+/–^* mice on a pure 129/SvJ background show normal central (Supplemental Figure 1; supplemental material available online with this article; https://doi.org/10.1172/jci.insight.184231DS1) and peripheral (Supplemental Figure 2) chemoreflexes at between 3 and 7 weeks of age. Together, these results suggest that Nav1.1 deficiency in the absence of seizures does not result in a respiratory phenotype but may predispose the respiratory system to disfunction following even modest seizure activity. These results also support the possibility that altered respiratory function can serve as an early indicator for increased SUDEP risk.

### Respiratory phenotype of juvenile and adult Scn1a^+/–^ mice on a mixed background.

**Figure 2:**
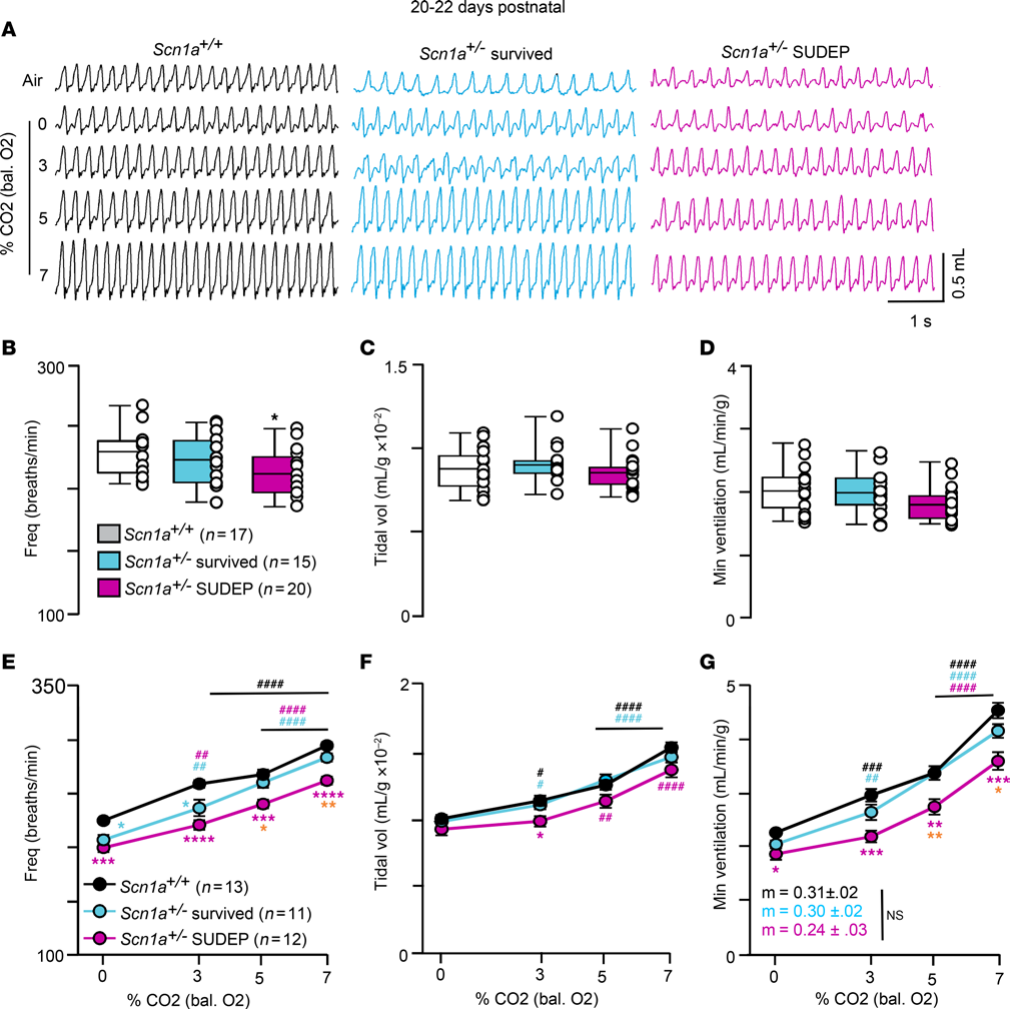
Three-week old *Scn1a^+/–^* mice that die prematurely show a pronounced central chemoreceptor deficit. *Scn1a^+/–^* mice begin to develop seizures and die at approximately 3 weeks of age (29). Therefore, for this analysis, we separated *Scn1a^+/–^* mice into 2 cohorts based on whether or not they died within 5 days of this experiment; these groups are termed *Scn1a^+/–^* survived and *Scn1a^+/–^* SUDEP. (**A**) Traces of respiratory activity from *Scn1a^+/+^* (*n* = 17), *Scn1a^+/–^* survived (*n* = 15), and *Scn1a^+/–^* SUDEP (*n* = 20) mice during exposure to room air and graded increases in CO_2_ (balance O_2_). (**B**–**D**) Summary data plotted as mean ± maximum/minimum show that *Scn1a^+/–^* SUDEP mice have a lower respiratory frequency (**B**) but otherwise similar tidal volume (**C**) (*P* = 0.82) and minute ventilation (**D**) (*P* = 0.1124) as compared with control or *Scn1a^+/–^* survived mice under room air conditions (1-way ANOVA followed by Tukey’s multiple-comparison test). (**E** and **F**) Summary data plotted as mean ± SEM of frequency (**E**) (*P* < 0.0001), tidal volume (**F**) (*P* = 0.055) and minute ventilation (**G**) (*P* = 0.0008) show that *Scn1a^+/–^* SUDEP mice have a reduced respiratory response to graded increases in CO_2_ that is mediated primarily by a blunted frequency response. Means were compared using repeated measures 2-way ANOVA followed by Tukey’s or Dunnett’s multiple-comparison test and slopes of minute ventilation 0%–7% CO_2_ responses were compared using 1-way ANCOVA. Orange asterisks represent differences between *Scn1a^+/–^* cohorts. *P < 0.05, **P < 0.01, ***P < 0.001, ****P < 0.0001 with Tukey’s multiple-comparison test for differences between genotypes; ^#^*P* < 0.05, ^##^*P* < 0.01, ^###^*P* < 0.001, ^####^*P* < 0.0001 with Dunnett’s multiple-comparison test for within-genotype differences from control.

To determine whether breathing problems in *Scn1a^+/–^* mice worsen with disease progression and contribute to SUDEP, we characterized baseline breathing and CO_2_ chemoreception in each genotype at 3 weeks of age. Furthermore, since the murine peripheral chemoreflex has matured by this stage of development (13), we also characterized the hypoxic ventilatory response. Note that 3-week-old mice of both genotypes showed comparable levels of metabolic activity across the 24-hour light/dark cycle (Supplemental Figure 3). Importantly, this time point also coincides with rapid onset of spontaneous seizures and SUDEP, so we divided the *Scn1a^+/–^* group into 2 cohorts based on whether animals succumb to SUDEP within 4 days of an experiment (these cohorts are termed *Scn1a^+/–^* survived and *Scn1a^+/–^* SUDEP mice). Consistent with the possibility that breathing problems worsen with disease progression and contribute to mortality, we found that *Scn1a^+/–^* SUDEP mice exhibit diminished breathing under baseline conditions and in response to hypercapnia (Figure 2A) and hypoxia. For example, in room air *Scn1a^+/–^* SUDEP mice (*n* = 20) show a reduced respiratory frequency compared with control (*n* = 17) or *Scn1a^+/–^* survived (*n* = 15) mice (*F*_2,49_ = 4.310, *P* = 0.016) (Figure 2B). Other baseline parameters, including tidal volume (*P* = 0.8176) (Figure 2C) and minute ventilation (*P* = 0.1124) (Figure 2D), were similar between groups. *Scn1a^+/–^* SUDEP mice also failed to increase respiratory frequency in response to graded increases in CO_2_ (Figure 2E) and despite the otherwise normal tidal volume response to high CO_2_ (Figure 2F), this resulted in diminished minute ventilation at both moderate and high levels of CO_2_ (*F*_2,33_ = 17.4, *P* = 0.008) (Figure 2G). We also noted that both cohorts of *Scn1a^+/–^* mice show diminished respiratory frequency under 100% O_2_ conditions compared with control (*Scn1a^+/–^* survived *F*_2,33_ = 29.33, *P* = 0.02 and *Scn1a^+/–^* SUDEP, *P* = 0.0006) (Figure 2E). Since breathing pure O_2_ will inhibit peripheral chemoreceptor drive, this finding suggests enhanced peripheral chemoreceptor drive helps both groups of *Scn1a^+/–^* mice maintain normal minute ventilation under room air conditions.

**Figure 3:**
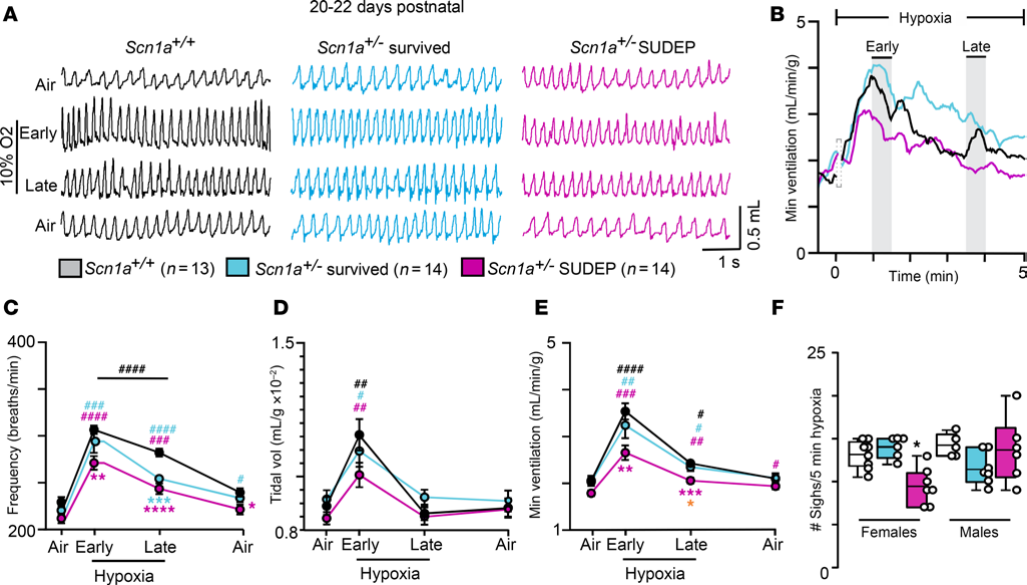
Three-week old *Scn1a^+/–^* SUDEP mice show a blunted hypoxic ventilatory response. (**A**) Traces of respiratory activity from 3-week-old *Scn1a^+/+^* (*n* = 13), *Scn1a^+/–^* survived (*n* = 14), and *Scn1a^+/–^* SUDEP (*n* = 14) mice in room air or during the early (90 seconds of hypoxia) and late phase (3.5–4.5 minutes) of exposure to hypoxia (10% O_2_/95% N_2_). (**B**) Traces of minute ventilation show the time course of the hypoxic response for each genotype. Note the transition to hypoxia typically corresponded to brief behavior artifacts that were omitted from analysis. (**C**–**E**) Summary data from control and each *Scn1a^+/–^* mouse cohort show respiratory frequency (**C**), tidal volume (**D**), and minute ventilation (**E**) in air (before and 10 minutes after hypoxia) and during the early and late phase of exposure to hypoxia. *Scn1a^+/–^* SUDEP mice show a diminished minute ventilatory response to both early and late phases of the hypoxia response (*F*_2,38_ = 8.3; early *P* = 0.004; late *P* = 0.0007). This peripheral chemoreceptor deficit is primarily mediated by a blunted frequency response (**C**). (**F**) Summary data plotted as mean ± maximum/minimum show the number of sighs detected in control and *Scn1a^+/–^* mice of each sex during 5 minutes of hypoxia. Means were compared using repeated measures mixed effects model followed by Tukey’s or Dunnett’s multiple-comparison test. Orange asterisks represents differences between *Scn1a^+/–^* cohorts. *P < 0.05, **P < 0.01, ***P < 0.001, ****P < 0.0001 with Tukey’s multiple-comparison test for differences between genotypes; ^#^*P* < 0.05, ^##^*P* < 0.01, ^###^*P* < 0.001, ^####^*P* < 0.0001 with Dunnett’s multiple-comparison test for within-genotype differences from control.

To explore this possibility further, we characterized the hypoxic ventilatory response in 3-week-old mice of each genotype. Considering the hypoxic ventilatory response consists of an early excitatory response that peaks at approximately 1 minute, followed by a ventilatory reduction phase that reaches a nadir (above pre-hypoxic levels) at approximately 3 minutes in hypoxia (14), and since each phase of this response reflects different but interrelated mechanisms (14), respiratory activity during each phase of a 5-minute exposure to 10% hypoxia was analyzed separately. Despite the apparent compensatory role of peripheral chemoreceptor drive under baseline conditions, *Scn1a^+/–^* SUDEP mice failed to increase minute ventilation early during hypoxia exposure (2.6 ± 0.15 μL/min/g; *n* = 9) by an amount proportional to control (3.5 ± 0.17 μL/min/g; *n* = 10) (*F*_2,38_ = 8.3, *P* = 0.0036) but not different from *Scn1a^+/–^* survived mice (3.24 ± 0.27 mL/min/g, *n* = 6, *P* = 0.21) (Figure 3, A–E). This was also evidenced by a diminished slope of the minute ventilatory response to early hypoxia in control mice (*m* = 1.5 ± 0.2) compared with *Scn1a^+/–^* SUDEP mice (*m* = 0.92 ± 0.16, *P* = 0.0427). This respiratory deficit was mediated by diminished respiratory frequency (*Scn1a^+/–^* SUDEP 271.1 ± 7.3 bpm vs. control 306.6 ± 4.5 bpm; *F*_2,38_ = 12.17, *P* = 0.0029) but not tidal volume (*P* = 0.138) (Figure 3, C and D). *Scn1a^+/–^* SUDEP mice also showed diminished minute ventilatory output (2.1 ± 0.06 mL/min/g, *n* = 14) during the later phase of the hypoxic challenge compared with control (2.4 ± 0.06 mL/min/g, *n* = 13; *F*_2,38_ = 8.3; *P* = 0.0007) or *Scn1a^+/–^* survived mice (2.3 ± 0.09 mL/min/g, *n* = 14; *F*_2,38_ = 8.3, *P* = 0.03) (Figure 3E), due largely to attenuation of respiratory frequency (*F*_2,38_ = 12.17, *P* < 0.0001) (Figure 3, C and D).

Interestingly, hypoxia also elicits sigh activity to promote gas exchange (15), and while SUDEP tends to occur more frequently in men (16, 17), we found that 3-week-old *Scn1a^+/–^* SUDEP female mice showed fewer sighs (9.4 ± 0.8 sighs, *n* = 7) compared with age-matched male *Scn1a^+/–^* SUDEP mice (13.7 ± 1.5 sighs, *n* = 6) or control or *Scn1a*^–/+^ survived mice of either sex (*F*_5,33_ = 4.855, *P* = 0.0019) (Figure 3F). It should be noted that *Scn1a*^–/+^ mice that live to 5–7 weeks of age recover a normal hypoxic ventilatory response (Supplemental Figure 4), whereas their ventilatory response to CO_2_ remains diminished compared with control (Supplemental Figure 5). Together, these results identify a strong correlation between altered central and peripheral chemoreception and increased SUDEP propensity.

### RTN neurons are hyperexcitable in slices from Scn1a^+/–^ mice on a mixed background.

**Figure 4:**
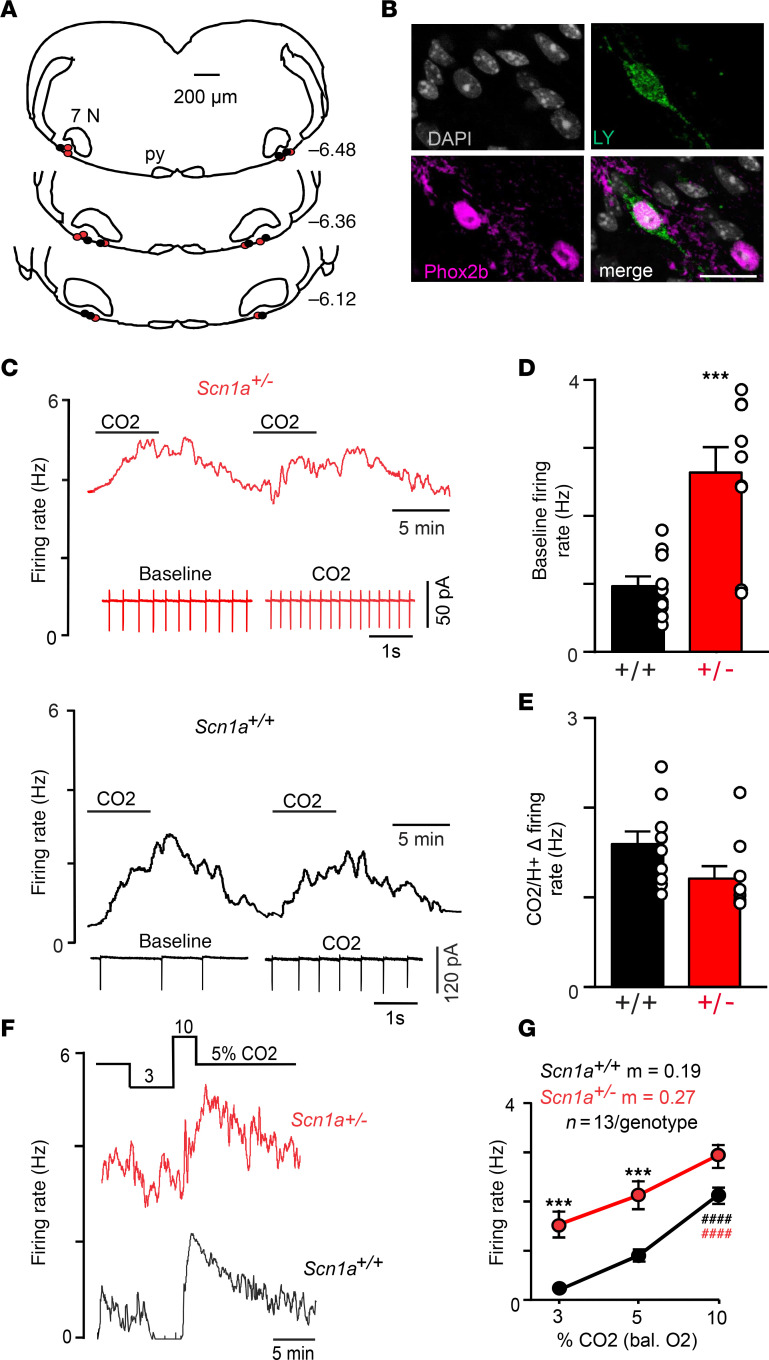
RTN neurons in slices from *Scn1a^+/–^* mice are hyperexcitable under control conditions and during hypocapnia. (**A**) Computer-assisted plot shows the location of RTN neurons from each genotype in the ventral parafacial region. Py, pyramidal tract; 7 N, facial motor nucleus. Numbers to the right of each section designate millimeters from bregma. (**B**) Double immunolabeling shows a Lucifer yellow–filled (LY-filled) CO_2_/H^+^-sensitive RTN neuron recorded in a slice from a *Scn1a^+/–^* mouse is Phox2b immunoreactive. DAPI was used to visualize the cell nucleus. Scale bar: 20 μm. We confirmed Phox2b immunoreactivity in RTN neurons from control (*n* = 10) and *Scn1a^+/–^* (*n* = 11) tissue. (**C**) Traces of firing rate and segments of holding current from RTN neurons in slices from control (black) and *Scn1a^+/–^* (red) mice show examples of spontaneous activity under control conditions (5% CO_2_, pH 7.3) and that neurons from both genotypes respond to 10% CO_2_ with a washable and repeatable increase in activity (pH 7.0). (**D** and **E**) Summary data show that RTN neurons in *Scn1a^+/–^* tissue exhibit high baseline activity (**D**) but respond to 10% CO_2_ by an amount similar to control neurons (**E**). (**F**) Traces of firing rate from RTN neurons in slices from control (black) and *Scn1a^+/–^* (red) mice show that exposure to 3% CO_2_ strongly inhibits control neurons but causes only a modest inhibition of neurons in *Scn1a^+/–^* tissue. (**G**) Summary data (*n* = 13/ genotype) show that RTN neurons in slices from *Scn1a^+/–^* mice are more excitable at 3% and 5% CO_2_. Means were compared using 2-way ANOVA followed by Šídák’s multiple-comparison test and slopes of neural activity between 3% and 10% CO_2_ were compared using 1-way ANCOVA. ***P < 0.001 for differences between genotypes; ^####^*P* < 0.0001 for genotype differences from control.

RTN neurons are a likely basis for chemoreceptor deficits in models of chronic epilepsy (18) and DS (8); therefore, we characterized functional properties of RTN neurons in brain slices from control and *Scn1a^+/–^* mice approximately 2 weeks of age. Considering Nav1.1 channels are highly expressed by ventral parafacial inhibitory neurons and to a lesser extent RTN neurons at this developmental time point (8), and since some inhibitory parafacial neurons limit activity of RTN neurons during hypocapnia (low CO_2_/H^+^) and augment output of the RTN during high CO_2_/H^+^ by a mechanism involving disinhibition (19), we expect RTN neurons in slices from *Scn1a*^+/–^ mice to show increased activity under low physiological levels of CO_2_ but otherwise respond normally to high CO_2_/H^+^ when activity of inhibitory neurons is suppressed. RTN neurons are identified in the cell-attached voltage clamp mode (V_hold_ –60 mV) by their location in the ventral parafacial region (Figure 4A) and their characteristic CO_2_/H^+^-dependent firing behavior (8); they are spontaneously active under control conditions (5% CO_2_, pH 7.3) and respond to 10% CO_2_ (pH 7.0) with at least a 0.8 Hz increase in firing rate. Chemosensitive RTN neurons also have been shown to express the transcription factor Phox2b (19–21); therefore, at the end of each experiment, we filled most recorded cells with Lucifer yellow for later immunohistochemical confirmation of Phox2b expression (Figure 4B). Consistent with expectations, RTN neurons in slices from *Scn1a^+/–^* mice showed increased activity under control conditions (*Scn1a^+/–^* 2.6 ± 0.3 Hz vs. *Scn1a^+/+^* 0.9 ± 0.1 Hz; *T*_17_ = 4.4, *P* = 0.0004) (Figure 4, C and D) and during exposure to 3% CO_2_ as compared with neurons from control mice (*Scn1a^+/–^* 1.5 ± 0.3 Hz vs. *Scn1a^+/+^* 0.2 ± 0.1 Hz; *F*_1,72_ = 45.23, *P* = 0.0002) (Figure 4, F and G). Also as predicted, RTN neurons in slices from each genotype showed comparable responses to 10% CO_2_ (CO_2_/H^+^ Δ in firing rate: *Scn1a^+/–^* 1.2 ± 0.1 Hz vs. *Scn1a^+/+^* 1.6 ± 0.1 Hz; *T*_17_ = 1.922, *P* = 0.078) (Figure 4E) and this resulted in a similar CO_2_/H^+^ response profile between 3% and 10% CO_2_ (slope: *Scn1a^+/–^* 0.19 ± 0.05 vs. *Scn1a^+/+^* 0.27 ± 0.02) (Figure 4G). These results show that disruption of RTN neural function precedes manifestation of respiratory symptoms by approximately 1 week.

### Pharmacological activation of Nav1.1 rescues RTN neural activity deficits by promoting inhibitory synaptic input.

**Figure 5:**
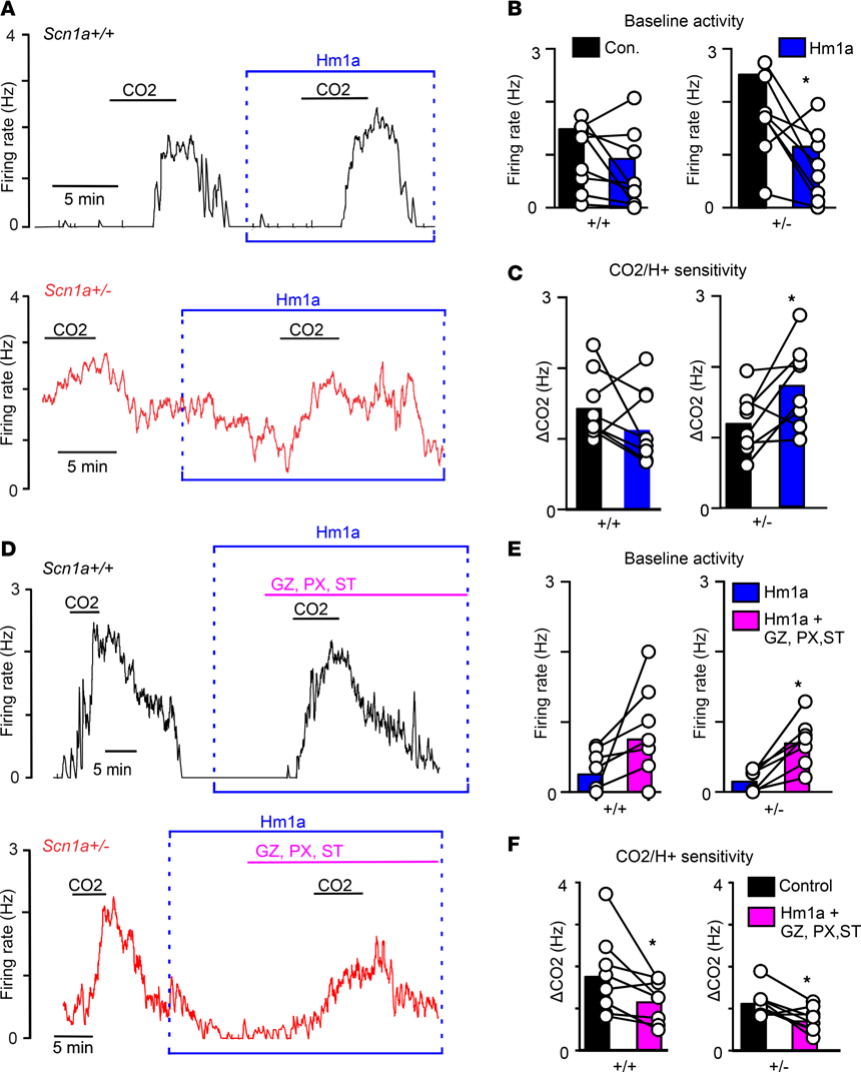
Hm1a normalizes baseline activity and increases hypercapnic sensitivity of RTN neurons by increasing inhibitory synaptic drive. (**A**–**C**) Representative traces of firing rate from RTN neurons in slices from control (black) and *Scn1a^+/–^* (red) mice (**A**) and summary data show that bath application of Hm1a (50 μM) decreased baseline activity (**B**) and increased the firing response to 10% CO_2_ (**C**) of RTN neurons in slices from *Scn1a^+/–^* mice. In contrast to this, Hm1a minimally affected baseline activity and CO_2_/H^+^ sensitivity of RTN neurons in slices from control mice (**A**–**C**). (**D**–**F**) Traces of firing rate from RTN neurons in slices from control (black) and *Scn1a^+/–^* (red) mice (**D**) and summary data show that application of gabazine (GZ; 50 μM), picrotoxin (PX; 50 μM), and strychnine (ST; 1 μM) to inhibit GABA_A_ and glycine receptors effectively reversed the effects of Hm1a on baseline activity (**E**) and CO_2_ sensitivity (**F**) of RTN neurons in slices from *Scn1a^+/–^* mice. Comparisons were made using a paired *t* test within genotypes and an unpaired *t* test across genotypes. *P < 0.05.

The above results are consistent with the possibility that diminished Nav1.1 function preferentially suppresses activity of inhibitory neurons early in development (22). Based on this, we wanted to determine whether targeted activation of Nav1.1 channels can rescue activity deficits exhibited by RTN neurons in *Scn1a^+/–^* mice. Consistent with expectations, we found that bath application of Hm1a (50 nM) — a compound that selectively increases Nav1.1 channel activity by decreasing voltage-dependent inactivation (23) — decreased baseline activity of RTN neurons in slices from *Scn1a^+/–^* mice by 52% to a level similar to control neurons (*Scn1a^+/–^* Hm1a 0.8 ± 0.2 Hz vs. *Scn1a^+/+^* 1.0 ± 0.2 Hz; *T*_15_ = 0.73, *P* = 0.47) (Figure 5, A and B). We also found that Hm1a increased the hypercapnic response of RTN neurons in slices from *Scn1a^+/–^* mice by 57% (*T*_7_ = 2.6, *P* = 0.03) (Figure 5, A and C). Conversely, Hm1a had negligible effect on baseline activity (*T*_8_ = 1.8, *P* = 0.1124) or CO_2_/H^+^ sensitivity (*T*_8_ = 1.5, *P* = 0.17) of RTN neurons in slices from control mice (Figure 5, A–C). Although not shown, we confirmed that bath application of Hm1a decreased the spontaneous inhibitory postsynaptic current interevent interval from 8.0 ± 2.5 seconds to 4.8 ± 2.1 seconds (*T*_3,4_, *P* = 0.04) but with no change in amplitude (*T*_2,4_, *P* = 0.09). These results show that Hm1a increased synaptic inhibition of RTN neurons in slices from *Scn1a^+/–^* mice. Together, these results suggest Hm1a preferentially rescues contributions of inhibitory synaptic drive to baseline activity and CO_2_/H^+^-dependent disinhibition of RTN neurons.

To further test this possibility, we determined whether the effects of Hm1a on activity of RTN neurons in slices from *Scn1a^+/–^* mice could be reversed by bath application of a cocktail of GABA_A_ (50 μM each of gabazine and picrotoxin) and glycine (1 μM strychnine) receptor blockers. Consistent with expectations, we found that RTN neurons in slices from *Scn1a^+/–^* mice in the continued presence of Hm1a (50 μM), subsequent bath application of cocktail of inhibitory receptor blockers increased baseline activity (Hm1a 0.17 ± 0.1 Hz vs. cocktail 0.71 ± 0.1 Hz; *T*_6_ = 5.068, *P* = 0.002) (Figure 5, D and E) and decreased the hypercapnic response (Hm1a 1.1 ± 0.11 Hz vs. cocktail 0.73 ± 0.10 Hz; *T*_6_ = 2.8, *P* = 0.03) (Figure 5, D and F). Control neurons did not show a marked increase in baseline activity following blockade of inhibitory receptors in the presence of Hm1a (*P* = 0.068) (Figure 5E). However, consistent with the possibility that disinhibition contributes to RTN chemoreception (24), we found that the firing response of control neurons to 10% CO_2_ was blunted in the cocktail of inhibitory receptor blockers plus Hm1a (control 1.8 ± 0.3 Hz vs. cocktail 1.2 ± 0.17 Hz; *T*_7_ = 2.5, *P* = 0.04) (Figure 5F). These results suggest Hm1a normalizes baseline activity and CO_2_/H^+^ sensitivity in part by increasing inhibitory synaptic drive of RTN neurons.

### Pharmacological activation of Nav1.1 increases repetitive firing capacity of RTN neurons.

**Figure 6:**
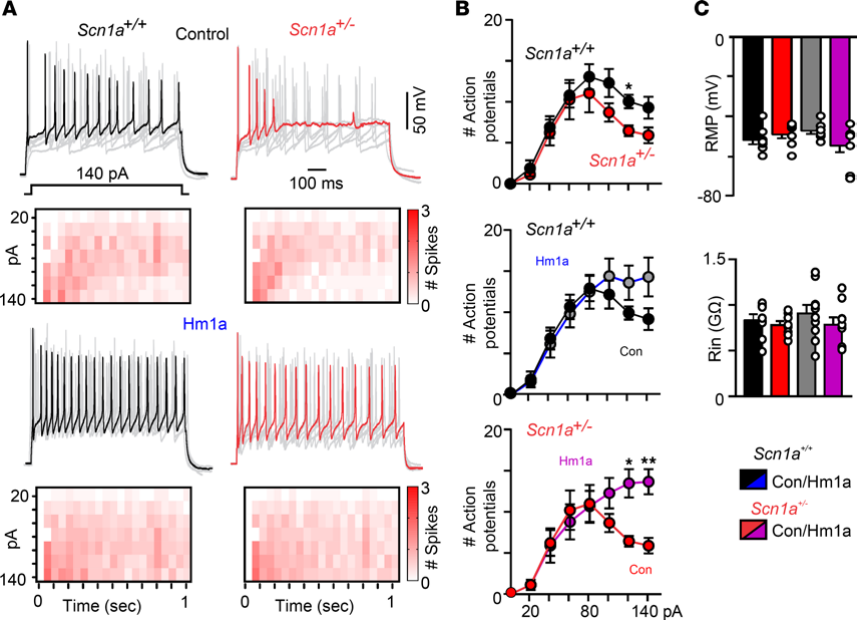
Hm1a improves the ability of RTN neurons in slices from control and *Scn1a^+/–^* mice to maintain repetitive firing behavior during sustained depolarization. (**A**) Segments of membrane potential and summary data plotted as spike discharge heatmaps (30-ms bins) from RTN neurons in slices from *Scn1a^+/+^* (black, *n* = 8) and *Scn1a^+/–^* (red, *n* = 9) mice during depolarizing current injections (20 to 140 pA; 1-second duration) from a holding potential of –80 mV before and 10 minutes following bath application of Hm1a (50 μM). (**B**) Summary data plotted as mean ± SEM number of action potentials evoked by depolarizing current injection shows that under control conditions RTN neurons in slices from both genotypes have a diminished capacity to maintain firing during larger step depolarizations, with RTN neurons in slices from *Scn1a^+/–^* mice being significantly diminished compared with control during +120 pA steps. Hm1a improves sustained firing behavior of RTN neurons in slices from *Scn1a^+/–^* mice at +120 pA (*F*_7,119_ = 5.3) and +140 pA but not *Scn1a^+/+^* neurons (*F*_7,112_ = 2.5, *P* = 0.48). Comparisons were made using 2-way ANOVA with Šídák’s multiple-comparison test. (**C**) Summary data (plotted as mean ± SEM) show that RTN neurons in slices from *Scn1a^+/+^* and *Scn1a^+/–^* mice have similar resting membrane potential and input resistance (measured during –60 pA steps) under control conditions and during incubation in Hm1a. *P < 0.05, **P < 0.01.

RTN neurons also express Nav1.1 channels at this stage of development (8, 19), so we wanted to determine whether Hm1a improves the ability of RTN neurons to maintain spike discharge during sustained depolarizations. We recorded repetitive firing behavior of RTN neurons in slices from each genotype in whole-cell current-clamp mode during duration step (1 second) depolarizations (20–140 pA; Δ 20 pA) from a holding potential of –80 mV. This hyperpolarized holding potential was used to maximize the proportion of Nav1.1 channels available to contribute to firing behavior. We found that properties (threshold, spike amplitude, and maximum rate of depolarization) of the first spike elicited by a +120 pA depolarizing step was similar between genotypes (Supplemental Figure 6) and showed similar repetitive firing responses to modest depolarizing current injections (Figure 6, A and B). However, RTN neurons in slices from *Scn1a^+/–^* mice were more prone to depolarizing block during larger sustained depolarizing current steps compared with RTN neurons in control tissue. For example, the number of spikes elicited by a +120 pA current step (1 second) was 10 ± 0.8 for *Scn1a^+/+^* controls (*n* = 8) compared with 6.4 ± 0.6 for *Scn1a^+/–^* (*n* = 9; *F*_7,105_ = 0.89, *P* = 0.03), but during larger depolarizations RTN neurons from both genotypes showed pronounced spike amplitude and frequency decrement consistent with depolarizing block (Figure 6, A and B). We also found that RTN neurons in slices from *Scn1a^+/–^* mice have a limited ability to respond to 1-second-long trains of depolarizing pulses (140–200 pA) when delivered at high frequency. For example, RTN neurons in slices from each genotype showed a similar low failure rate (depolarizing stimulus that does not elicit an action potential) when stimulated at between 10 and 20 Hz. However, RTN neurons in slices from *Scn1a^+/–^* mice failed more frequently than neurons in control tissue at 50 Hz stimulation (Supplemental Figure 7). Considering Nav1.1 channel inactivation is an important determinant of depolarizing block (25), and since Hm1a decreases Nav1.1 channel inactivation (23), these results provide clarity regarding the preferential effects of Hm1a on RTN neurons in *Scn1a^+/–^* tissue compared with control. Also consistent with this possibility, we show that bath application of Hm1a rescued the ability of RTN neurons in *Scn1a^+/–^* tissue to maintain spike discharge during large, sustained depolarizations. For example, Hm1a increased the number of spikes elicited by RTN neurons in slices from *Scn1a^+/–^* mice during a +140 pA current step (1 second) from 5.9 ± 0.95 to 13.7 ± 1.5 (*F*_7,119_ = 5.3, *P* = 0.0046) (Figure 6, A and B). Passive electrical properties, including resting membrane potential (*Scn1a^+/+^* –52 ± 2.1 mV vs. *Scn1a^+/–^* –48 ± 1.4 mV; *P* > 0.05) and input resistances (*Scn1a^+/+^* 831 ± 66 MΩ vs. *Scn1a^+/–^* 781 ± 42 MΩ; *P* > 0.05), were similar between genotypes (Figure 6C).

## Discussion

SUDEP is the leading cause of mortality in epilepsy. Despite this, there are no biomarkers of SUDEP, so identifying at-risk patients remains a major challenge. Results presented here address this need by identifying altered baseline breathing and diminished central and peripheral chemoreception as early indicators of mortality in a mouse model of DS. Considering baseline breathing and the ventilatory response to CO_2_ are readily assessable parameters in the clinic with minimal risk to the patient (26), we believe this metric will serve as a reliable biomarker of SUDEP risk. Our results also identify RTN neurons as a likely basis for breathing problems in DS and show that a therapeutic approach designed to decrease Nav1.1 inactivation can reestablish inhibitory control of RTN neurons and rescue cellular activity deficits.

Seizures typically originate in the cortex, and it is not clear whether brainstem respiratory function is compromised directly by loss of *Scn1a* or indirectly by descending seizure activity (27). Our evidence that modest breathing problems are detectable in 2-week-old *Scn1a^+/–^* mice, a time point that precedes obvious seizure activity (*Scn1a^+/–^* mice on a 50% C57BL/6J:50% 129/SvJ background begin to show spontaneous seizures of varying frequency between 20 and 28 days of age) (28, 29), suggests loss of Nav1.1 directly impacts brainstem respiratory centers. However, we also found that *Scn1a*-haploinsufficient mice on a pure 129/SvJ background, which do not exhibit seizures, also did not present detectable breathing problems. Based on these observations, we speculate that Nav1.1 deficiency in the absence of seizures does not result in abnormal breathing but rather may predispose the respiratory system to disfunction when even modest seizures occur. The initial respiratory phenotype of *Scn1a^+/–^* mice involves a blunted response to moderate but not high levels of CO_2_. This finding is consistent with evidence that *Scn1a* transcripts are preferentially expressed by inhibitory parafacial neurons early in development (8) where Nav1.1 serves to maintain interneuron activity, including somatostatin-expressing (SST-expressing) neurons that normally limit activity of RTN neurons under low/moderate CO_2_/H^+^ levels but not during high CO_2_ when inhibitory parafacial neurons are less active (19). Therefore, under high CO_2_ conditions, RTN neurons may be disinhibited and consequently less sensitive to network consequences of loss of Nav1.1 channels from inhibitory neurons. In line with this, previous work showed that conditional expression of an *Scn1a* loss-of-function variant (A1783V) in all inhibitory neurons disrupted activity of RTN neurons in a manner consistent with disinhibition (8). At the level of RTN neurons, SST-expressing neurons are the only population of inhibitory neurons in the ventral parafacial region that influence respiratory behavior (19); we speculate that loss of Nav1.1 from ventral parafacial SST-expressing neurons contributes to breathing problems in *Scn1a^+/–^* mice. However, it is important to recognize that global *Scn1a* deficiency may impact breathing at any level of the respiratory circuit; therefore, other respiratory centers may contribute. Consistent with this, 3-week-old *Scn1a^+/–^* mice show a diminished ventilatory response to hypoxia. Carotid body glomus cells express *Scn1a* (30) and so may be directly impacted in this mouse model. It is also worth noting that RTN neurons are a key integration center for peripheral chemotransduction (31), and since loss of Nav1.1 function disrupts activity of RTN neurons (8), it is possible RTN neurons are a common substrate for abnormal central and peripheral chemoreflexes in *Scn1a^+/–^* mice. These findings are the first evidence to our knowledge implicating compromised peripheral chemoreception in DS-associated SUDEP, and together these results support the utility of disordered breathing as a biomarker of SUDEP.

If DS results from loss of Nav1.1 channel function in inhibitory neurons, then an approach that selectively increases Nav1.1 channel activity is expected to rescue features associated with this disease. Consistent with this, we show that bath application of Hm1a, which selectively increases Nav1.1 conductance by decreasing voltage-dependent inactivation, rescued activity deficits of RTN neurons by a GABA/glycine-dependent mechanism. These results are consistent with evidence that intracranial delivery of Hm1a reduced seizure susceptibility and improved mortality in *Scn1a^+/–^* mice. Interestingly, we also found that Hm1a had negligible effect on RTN neurons in slices from wild-type mice. This is consistent with the relatively modest role of Nav1.1 channels in regulation of excitatory neurons relative to inhibitory neurons (32). This is also consistent with evidence from the hippocampus showing that Hm1a had minimal impact on firing properties of GABAergic interneurons in slices from wild-type mice (23). Based on this, we speculate that inhibitory neurons in wild-type mice (with the full complement of Nav1.1 channels) have a lower proportion of Nav1.1 channels in the inactive state under resting conditions compared with inhibitory neurons in *Scn1a^+/–^* tissue. The ability of Hm1a to augment Nav1.1 channel function preferentially in *Scn1a*-deficient cells is therapeutically advantageous because expression of *Scn1a* can vary between cells — a factor contributing to phenotype variability associated with the same *Scn1a* mutation (33) — and overactivation of Nav1.1 can be pathogenic (34, 35). However, the therapeutic utility of Hm1a remains limited because it does not cross the blood-brain barrier and systemic application of this drug may disrupt peripheral pain sensitivity (36). Also, efforts to develop gene therapies designed to increase Nav1.1 expression specifically in inhibitory neurons promise improved seizure control with less unwanted side effects compared with current pharmacological approaches that drive global increases in GABA signaling (37, 38).

In sum, our results (a) identify disordered breathing as an early biomarker of SUDEP in a mouse model of DS, (b) establish the RTN as a likely basis for breathing problems in DS, and (c) show that targeted activation of Nav1.1 can rescue activity of RTN neurons in *Scn1a^+/–^* mice.

## Methods

### Sex as a biological variable

Our study characterized respiratory behavior in age-matched male and female *Scn1a^+/–^* and control mice. Primary outcome measures were compared between sexes and differences are reported in the text.

### Animals

Animals were housed in a 12-hour light/dark cycle with unlimited access to normal chow at weaning age. Female 129/SvJ *Scn1a^+/–^* mice (The Jackson Laboratory, strain 037107) were crossed with male C57BL/6J (The Jackson Laboratory, strain 000664) mice to produce *Scn1a^+/+^* and *Scn1a^+/–^* on a 50% C57BL/6J:50% 129/SvJ mixed background. Animals had unlimited access to normal chow and an enrichment hutch. We did not observe sex differences in minute ventilation in 21-day-old mice (*Scn1a^+/+^*, *Scn1a^+/–^* survived, and *Scn1a^+/–^* SUDEP) under room air conditions (*P* > 0.05) or during hypercapnia 7% (*P* > 0.05) or late hypoxia (*P* > 0.05), so male and female mice were pooled for all comparisons unless otherwise stated. Mice that exhibited seizure activity within 2 hours of an experiment were excluded from analysis.

### Unrestrained whole-body plethysmography

#### Adult mice.

Respiratory activity was measured using a whole-body plethysmograph system and ventilated at 1.1 L/min (Data Scientific International; DSI). Chamber temperature and humidity were continuously monitored and used to correct tidal volume on a breath-by-breath basis. Mice were individually placed into the chamber and allowed 1 hour to acclimate prior to the start of an experiment. Respiratory activity was recorded using Ponemah 5.32 software (DSI) for a period of 20 minutes in room air followed by exposure to graded increases in CO_2_ from 0% to 7% CO_2_ against a hyperoxic background (balance O_2_) to minimize peripheral chemoreceptor drive. On separate days, we also characterized the ventilatory response to 10% O_2_ (balance N_2_). All plethysmography experiments were video recorded and sections of data containing behavior artifacts were excluded from analysis. Parameters of interest include respiratory frequency (breaths/minute), tidal volume (*V*_T_, measured in mL; normalized to body weight and corrected to account for chamber temperature, humidity, and atmospheric pressure), and minute ventilation (*V*_E_, mL/min/g). A 20-second period of relative quiescence after 4–5 minutes of exposure to each condition was selected for all analysis. An apneic event was defined as 3 or more missed breaths that terminate when near-normal breathing frequency resumes. Sighs were identified based on their characteristic large amplitude (2 × tidal volume). Note that during the transition from air to hypoxia corresponded to a brief period of hyperlocomotor activity that was excluded from analysis.

#### Mouse pups.

A pup whole-body plethysmograph chamber was used to measure respiratory activity in mouse pups (12–14 days of age) (Buxco/DSI). Pups were placed on a warming stage with the plethysmography chamber that was maintained at 31.5°C to minimize loss of body temperature, while at the same time ensuring a robust signal-to-noise ratio. Chamber temperature and humidity were monitored and used to correct tidal volume on a breath-by-breath basis. Pup ventilatory responses to high CO_2_ were characterized as described above for adult animals.

### Brain slice preparation and cellular electrophysiology

Slices containing the RTN were prepared as previously described (8, 19). In short, *Scn1a^+/+^* (control) and *Scn1a^+/–^* mouse pups (9–11 days postnatal, mixed sex) were anesthetized by administration of ketamine (375 mg/kg, i.p.) and xylazine (25 mg/kg; i.p.) then rapidly decapitated; brainstems were removed and transverse brainstem slices (200–220 μm) were cut using a microslicer (DSK 1500E, Dosaka) in ice-cold modified Ringer’s solution containing (in mM): 260 sucrose, 3 KCl, 5 MgCl_2_, 1 CaCl_2_, 1.25 NaH_2_PO_4_, 26 NaHCO_3_, 10 glucose, and 1 kynurenic acid. Slices were incubated for 30 minutes at 37°C and followed by room temperature in normal Ringer’s solution containing (in mM): 130 NaCl, 3 KCl, 2 MgCl_2_, 2 CaCl_2_, 1.25 NaH_2_PO_4_, 26 NaHCO_3_, and 10 glucose. Both substituted and normal Ringer’s solutions were bubbled with 95% O_2_ and 5% CO_2_ (pH = 7.3).

Individual slices containing the RTN were transferred to a recording chamber mounted on a fixed-stage microscope (Olympus BX5.1WI) and perfused continuously (~2 mL/min) with a bath solution containing (in mM): 130 NaCl, 3 KCl, 2 MgCl_2_, 2 CaCl_2_, 10 HEPES, 10 glucose (equilibrated with 5% CO_2_; pH = 7.3). All recordings were made with an Axopatch 200B patch-clamp amplifier (Molecular Devices), digitized with a Digidata 1550B A/D converter (Molecular Devices) and recorded using ClampEx 11.0.3 software. Recordings were obtained at room temperature (~22°C) with patch electrodes pulled from borosilicate glass capillaries (Harvard Apparatus) on a 2-stage puller (P-97, Sutter Instrument) to a DC resistance of 5–7 MΩ when filled with pipette solution. In some experiments, RTN slices were treated with Hm1a (Alomone Labs, STH-601) alone or Hm1a plus gabazine (Tocris, SR 95531), picrotoxin (Tocris, 1128), and strychnine (Sigma-Aldrich, S0532).

Firing activity was measured in the cell-attached (seal resistance > 1 GΩ) voltage-clamp (V_hold_ −60 mV) configuration using a pipette solution containing (in mM): 125 K-gluconate, 10 HEPES, 4 Mg-ATP, 3 Na-GTP, 1 EGTA, 10 Na-phosphocreatine, 0.2% Lucifer yellow (pH 7.30). Electrophysiological data were acquired (50 kHz sampling rate and filtered at 10 kHz) and analyzed using Pclamp v11.0.3. software (Molecular Devices), and firing-rate histograms were generated by integrating action potential discharge in 10- to 15-second bins using CED Spike 5.0 software (Cambridge Electronic Design Limited). Once a CO**_2_**/H^+^-sensitive neuron was identified, we obtained whole-cell access and in current-clamp mode measured resting membrane potential and input resistance. Also, in whole-cell current-clamp mode, we characterized firing responses to depolarizing current steps (from +25 to +125 pA, Δ 20 pA, 1-second duration) from a holding potential of –80 mV. All whole-cell recordings had an access resistance of less than 20 MΩ. A liquid junction potential of –10 mV was corrected off-line.

### Immunohistochemistry

After recording, slices were fixed with 4% paraformaldehyde at least 24 hours at 4°C, washed in PBS (5 times, 5 minutes each), permeabilized in PBS/0.2% Triton X-100 (1× PBST; 2 times, 5 minutes each), and blocked in PBST/2% normal donkey serum for 2 hours. Slices were incubated overnight at room temperature with fresh blocking solution, goat anti-Phox2b antibody (1:150; R&D Systems, AF4940) and rabbit anti–Lucifer yellow antibody (1:450; Thermo Fisher Scientific, A-5750). Slices were washed the next morning (PBST; 5 times, 10 minutes each) and incubated for 2 hours in blocking solution with donkey anti-goat Alexa Fluor 647 (AB_2340436; Jackson ImmunoResearch, 705-605-003) and anti-rabbit Alexa Fluor 488 (AB_2313584; Jackson ImmunoResearch, 711-545-152) at 1:500. Slices were then washed in PBST and PBS (both 5 times, 10 minutes each), mounted on glass slides using Prolong Diamond with DAPI (Thermo Fisher Scientific), and imaged with a Leica SP8 confocal microscope.

### Comprehensive lab animal monitoring

Metabolic monitoring O_2_ consumption (VO_2_) and CO_2_ production (VCO_2_) was performed using comprehensive lab animal monitoring systems (CLAMS, Columbus Instruments). Adult mice approximately 3 week of age were individually housed on a 12-hour light/dark cycle in cages with a running wheel, regular bedding, and regular chow for 1 week before experimentation. Three days before metabolic assessment, each animal was placed in the CLAMS housing cage with metered water and waste collection. Mice were given 2 days to acclimate to the metabolic chamber; on the third day, all results were recorded for a continuous 24-hour period (Oxymax v5.54, Columbus Instruments). After data collection, results were exported and averaged per hour, only including times of no-wheel activity as assessed by an activity monitoring system within the CLAMS cage. Then, light and dark periods were determined and averaged per animal for statistical analysis. We focused our analysis on the respiratory exchange ratio (volume of CO_2_ produced/volume O_2_ consumed). Both sexes are equally represented in the data set.

### Statistics

All data sets were tested for normality using the Anderson-Darlington, D’Agostino & Pearson, Kolmogorov-Smirnov, and Shapiro-Wilk tests and outlier data points were identified using the Grubbs test and excluded from analysis. Normally distributed data were analyzed using paired or unpaired 2-tailed *t* test, 1-way or 2-way ANOVA followed by Tukey’s or Dunnett’s multiple-comparison test, or mixed effects ANOVA when applicable. Data sets that failed all 4 normality tests above were considered non-normally distributed and analyzed using the Mann-Whitney test. Also, the non-parametric analysis of covariance was used to compare slopes of linear regressions. The specific test used for each comparison is reported in the figure legend and all relevant values used for statistical analysis are included in the Results. Summary data are plotted as mean ± SEM along with individual data points; differences between means were considered significant when *P* was less than 0.05. The *P* values reported in the text represent differences between genotype effects based on the appropriate post hoc test. Box-and-whisker plots show the mean (line within each box), lower and upper quartiles (box bounds), and minimum and maximum values (whiskers). No outliers are shown.

### Study approval

All procedures were performed in accordance with the NIH *Guide for the Care and Use of Laboratory Animals* (National Academies Press, 2011) and the University of Connecticut Animal Care and Use Guidelines.

### Data availability

Raw data values for all main figures and supplemental figures are provided in the Supporting Data Values file, with clearly labeled tabs corresponding to each figure panel.

## Author contributions

BMM designed experiments, generated data, analyzed results, composed figures, edited the manuscript, and approved the final manuscript. DKM designed experiments, analyzed results, drafted the manuscript, and approved the final manuscript. ENS, CRS, and MLS generated data.

## Funding support

NIH, grant, to DKM.

NIH, grant, R01HL104101, to DKM.

NIH, grant, R01HL137094, to DKM.

NIH, grant, R21NS134132, to DKM.

NIH, grant, F31 NS120467, to BMM.

NIH, grant, F31 HL167553, to MLS.

Congenital Central Hypoventilation Syndrome (CCHS) network, grant, to DKM.

Simons Foundation Autism Research Bridge to Independence, grant, to CRS.

## Supplementary Material

Supplemental data

Supporting data values

## Version 1 09/09/2025

In-Press Preview

## Version 2 10/22/2025

Electronic publication
