# Supplementary material for: Developmental progression of respiratory dysfunction in a mouse model of Dravet syndrome
Source: JCI Insight. 2025 Sep 9;10(20):e184231. doi: 10.1172/jci.insight.184231 (PMC12581664; doi:10.1172/jci.insight.184231)
Supplement: Supplemental data [file jciinsight-10-184231-s115.pdf]

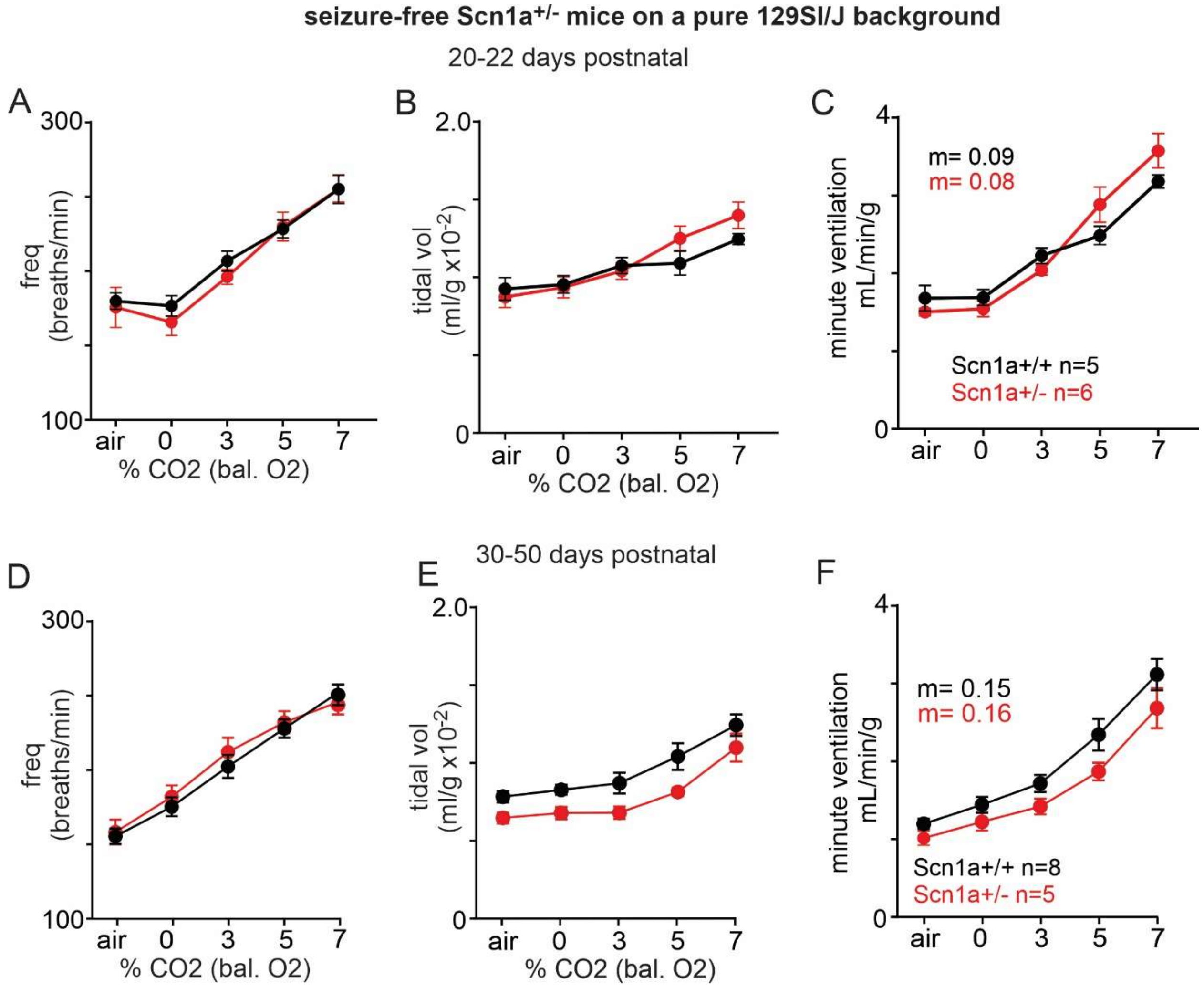

Supplemental Figure 1. Seizure-free *Scn1a*$^{+/-}$ mice on a pure 129SI/J background exhibit normal baseline breathing and central chemoreflex. A-C, summary plots of respiratory frequency (A, $p > 0.05$), tidal volume (B, $p > 0.05$) and minute ventilation (C, $p > 0.05$) show that ~2-week-old seizure free *Scn1a*$^{+/-}$ mice on pure 129SI/J background exhibit baseline respiratory activity and a ventilatory response to graded increases in CO_2 (0-7%, balance O_2) similar to control mice. **D-F**, summary plots of respiratory frequency (D, $p > 0.05$), tidal volume (E, $p > 0.05$) and minute ventilation (F, $p > 0.05$) show that ~4-7 week-old *Scn1a*$^{+/-}$ mice on pure 129SI/J background exhibit normal baseline respiratory activity and a ventilatory response to graded increases in CO_2 (0-7%, balance O_2). Means are compared using repeated measures two -way ANOVA followed by Sidak multiple comparison test and slopes of minute ventilation 0-7% CO2 responses are compared using one-way ANCOVA.

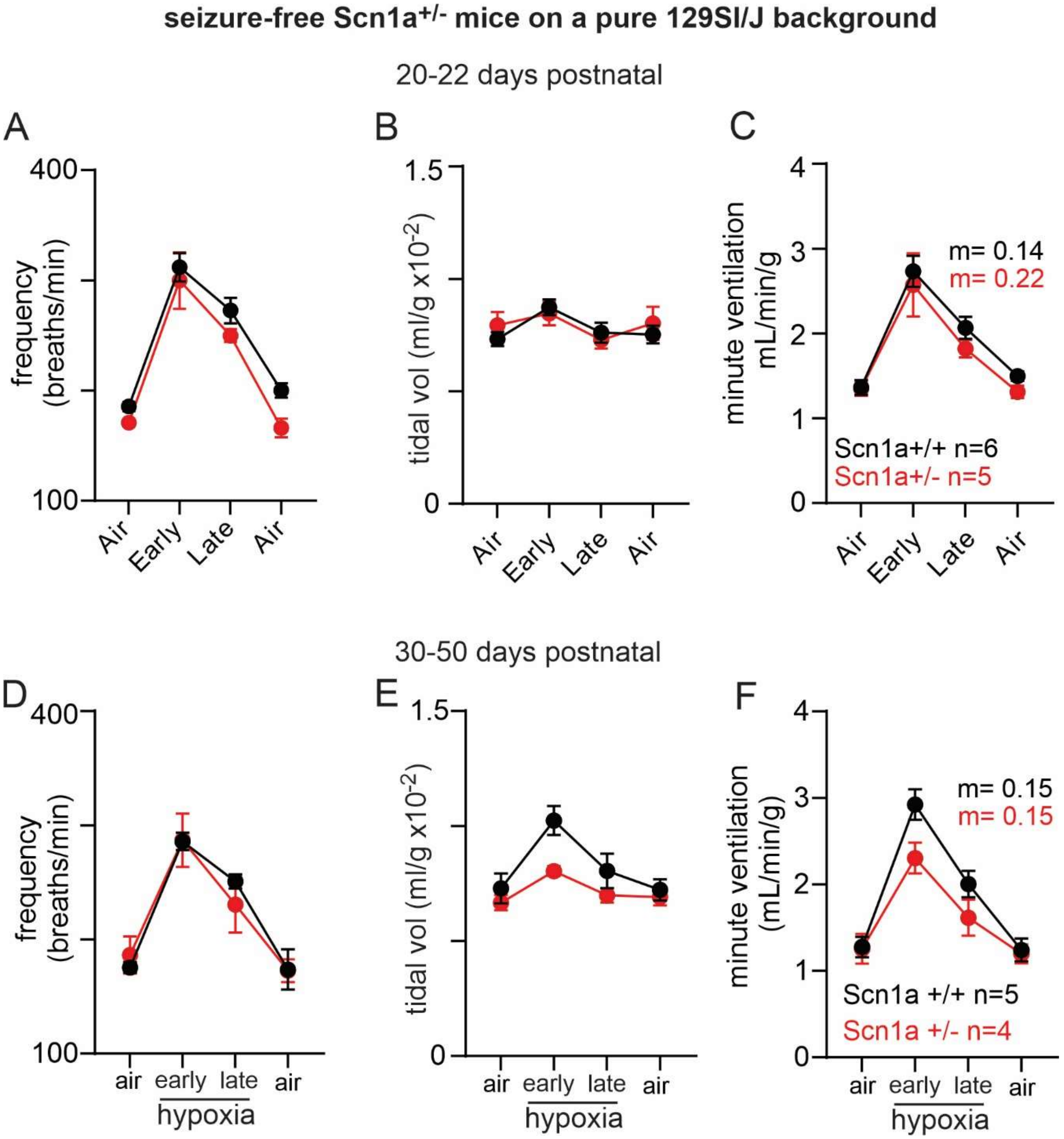

Supplemental Figure 2. Seizure-free *Scn1a*$^{+/-}$ mice on a pure 129SI/J background exhibit an normal hypoxic ventilatory response. **A-C**, summary plots show respiratory frequency (A), tidal volume (B) and minute ventilation (C, p >0.05) of ~2-week-old seizure free *Scn1a*$^{+/-}$ mice on pure 129SI/J background in air and during the early and late phase of exposure to hypoxia (10% O_2, 5 min). **D-F**, summary plots of respiratory frequency (D), tidal volume (E) and minute ventilation (F, p >0.05) show that ~4-7-week-old *Scn1a*$^{+/-}$ mice on pure 129SI/J background exhibit a normal ventilatory response to hypoxia. Means are compared using repeated measures mixed effects ANOVA followed by Sidak comparison test.

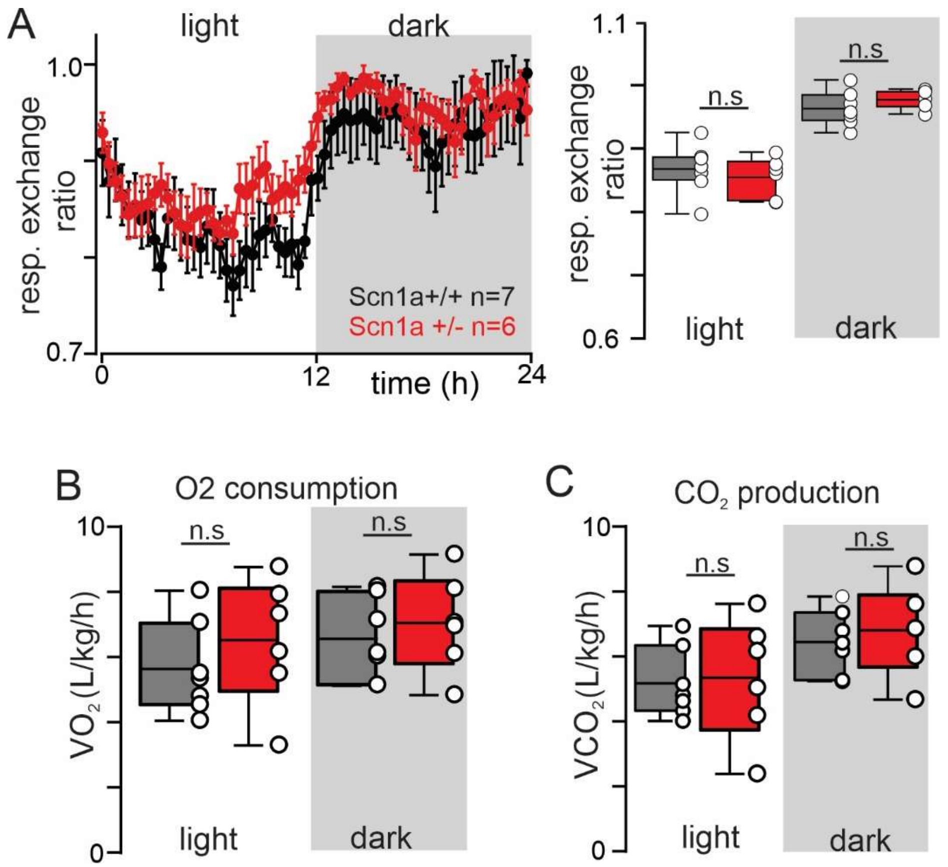

Supplementary Figure 3: *Scn1a*$^{+/+}$ and *Scn1a*$^{+/-}$ show similar baseline metabolic activity. We characterized baseline metabolic activity in adult (3 week old) *Scn1a*$^{+/+}$ (n= 7 mice) and *Scn1a*$^{+/-}$ mice (n=6) across a 24 hours light and dark cycle. This was performed by measuring the volume of CO_2 produced (VCO_2) relative to O_2 consumed (VO_2) and determining the respiratory exchange ratio (VCO_2/VO_2) under light/inactive and dark/active states. **A,** summary respiratory exchange ratio (RER) data plotted over time (left) and peak response during the light and dark cycles (right) show that *Scn1a*$^{+/+}$ and *Scn1a*$^{+/-}$ have similar levels during light ($p > 0.05$) and dark cycles ($p > 0.05$). **B**, summary data shows that *Scn1a*$^{+/+}$ and *Scn1a*$^{+/-}$ have similar baseline O_2 consumption during the light ($p > 0.05$) and dark cycles ($p > 0.05$). **C,** summary data show that *Scn1a*$^{+/+}$ and *Scn1a*$^{+/-}$ produce similar levels of CO_2 during the light ($p > 0.05$;) and dark cycles ($p > 0.05$). Baseline metabolic activity was compared using an unpaired two-way t-test.

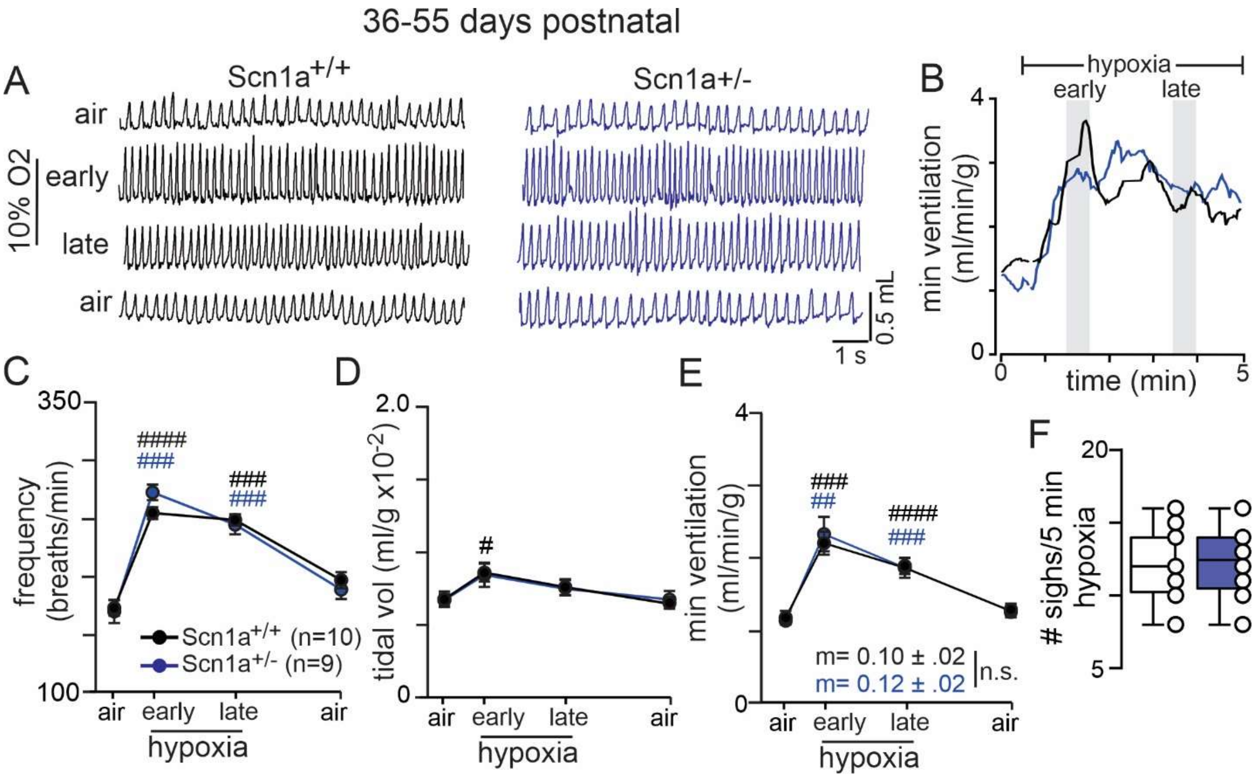

Supplementary Figure 4. ***Scn1a***$^{+/-}$ **survive mice show normal hypoxic ventilatory response at 5-7 weeks of age. A.** Traces of respiratory activity from a P35-P55 *Scn1a*$^{+/+}$ and *Scn1a*$^{+/-}$ survived mice in air and during early (90 seconds of hypoxia) and late phases (3.5-4.5 min) of hypoxia exposure (10% O_2 95 %N_2). **B**, Traces of minute ventilation show the time course of the hypoxic response for each genotype. Note the transition from air to hypoxia typically corresponded with brief behavior artifacts that were omitted from analysis. **C-E**, Summary data from control (n=10) and *Scn1a*$^{+/-}$ survive (n=9) mice show respiratory frequency (**C**), tidal volume (**D**) and minute ventilation (**E**) in air (before and 10 min. after hypoxia) and during the early and late phase of exposure to hypoxia. **F,** summary data plotted as mean ± maximum and minimum shows the number of sighs detected in control and *Scn1a*$^{+/-}$ mice (mixed sex) during 5 minutes of hypoxia. Means are compared using repeated measures mixed effects analysis followed by Sidaks or Dunnett's multiple comparison test. *, indicates difference between genotypes (Tukey's multiple comparison test); #, identifies within genotype differences from control (Dunnett's multiple comparison test). One symbol = $p < 0.05$, two symbols = $p < 0.01$, three symbols = $p < 0.001$, four symbols = $p < 0.0001$.

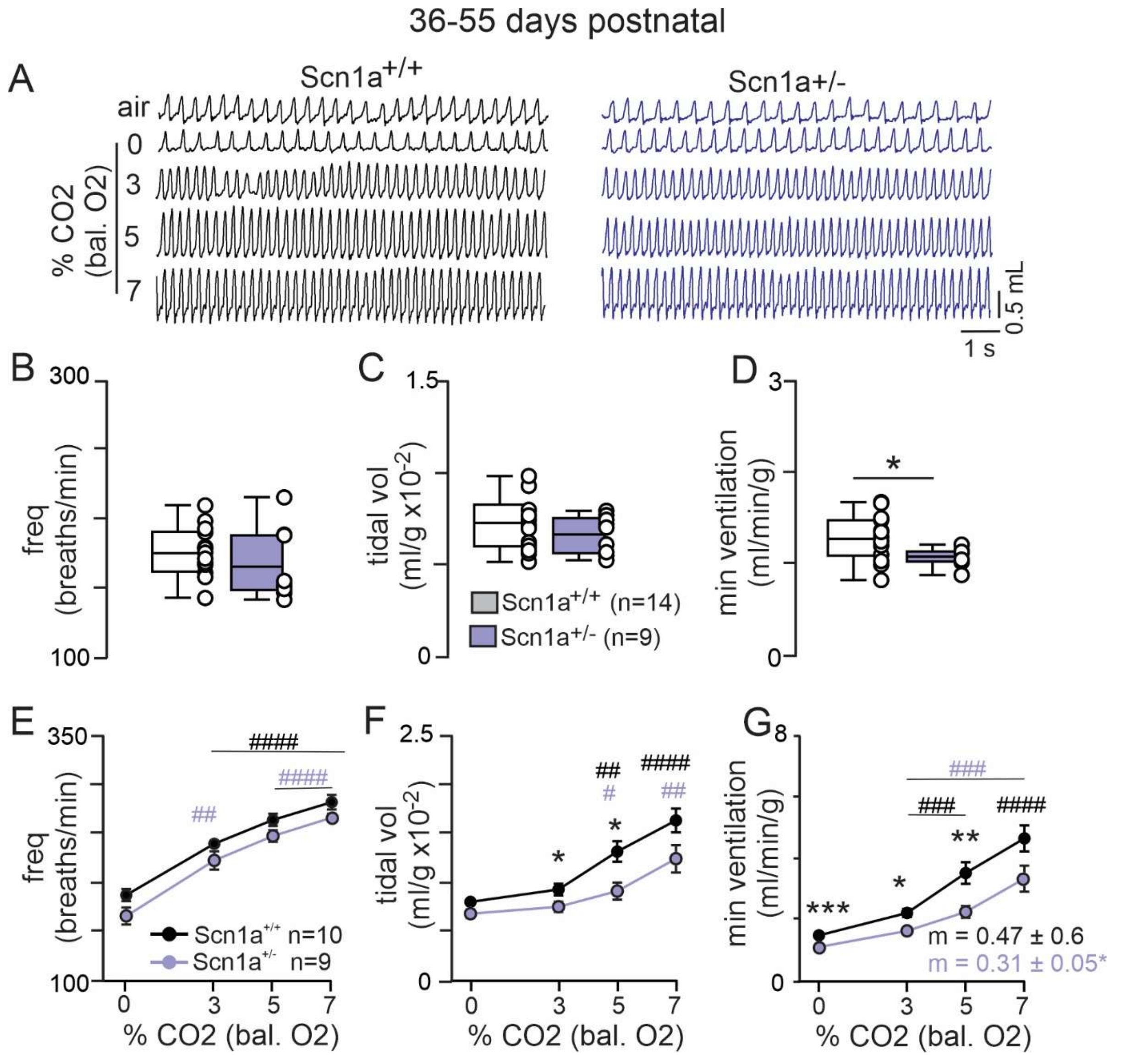

Supplemental Figure 5. Central chemoreceptor deficits persist in *Scn1a*$^{+/-}$ survived mice at 5-7 weeks of age. **A**, Traces of respiratory activity from a *Scn1a*$^{+/+}$ (n=14), *Scn1a*$^{+/-}$ survived (n=9) mice during exposure to room air and graded increases in CO_2 (balance O_2)**.** **B-D.** Summary data plotted as mean ± maximum/minimum show that *Scn1a*$^{+/-}$ survive mice have a lower minute ventilatory output in room air compared to control mice ($T_{18} = 2.6$, $p = 0.02$). **E-F,** Summary data plotted as mean ± SEM of frequency (**E**, p = 0.30), tidal volume (**F**, p= 0.036) and minute ventilation (**G**, p = 0.007) show that *Scn1a*$^{+/-}$ survive mice have a reduced respiratory response to graded increases in CO_2. Means are compared using repeated measures two-way ANOVA followed by Sidak's multiple comparison test or individual with a Welch t-test and slopes of minute ventilation 0-7% CO_2 responses are compared using one-way ANCOVA. *, indicates differences between genotypes; #, identifies within genotype differences from control (Dunnett's multiple comparison test). One symbol = $p < 0.05$, two symbols = $p < 0.01$, three symbols = $p < 0.001$, four symbols = $p < 0.0001$.

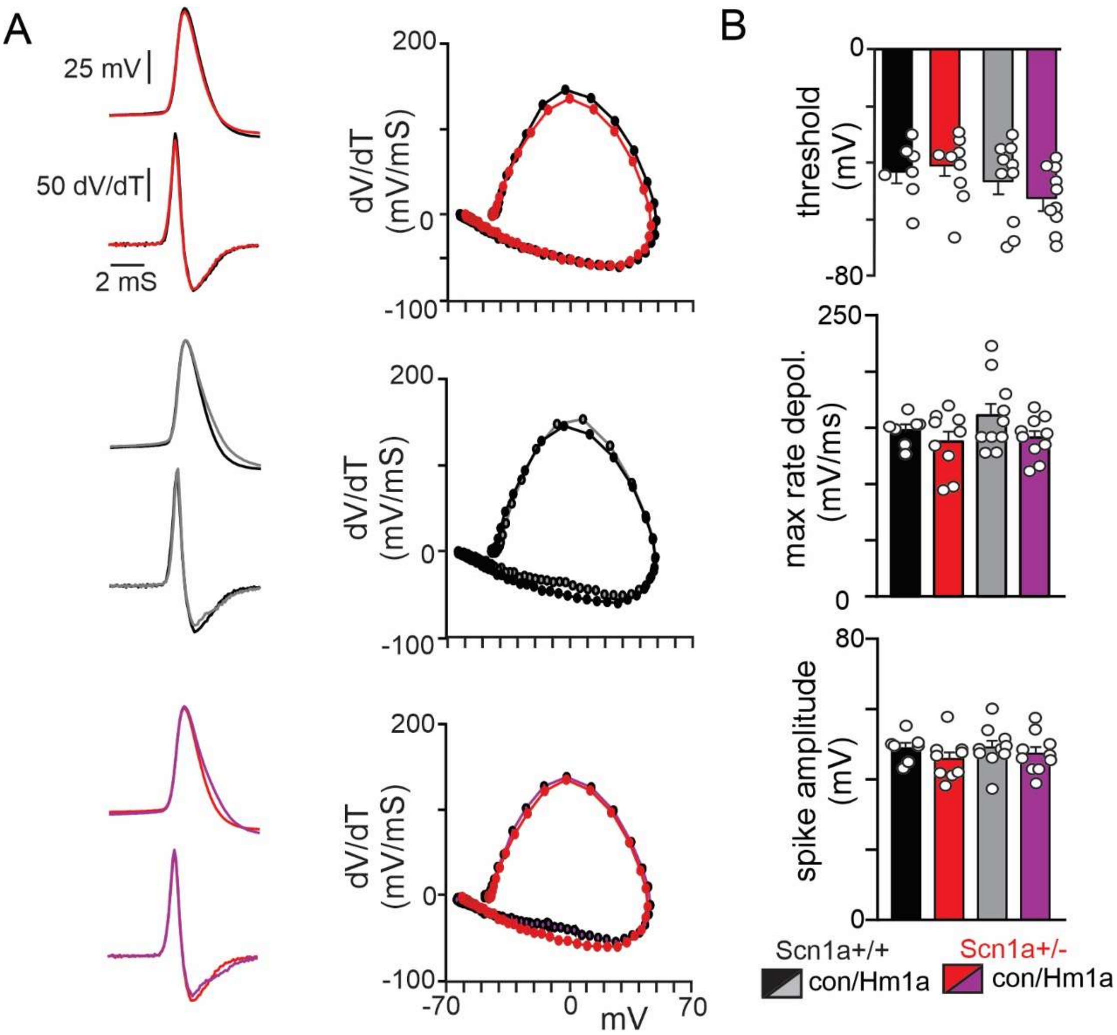

Supplementary Figure 6. RTN neurons in control and *Scn1a*$^{+/-}$ mice show similar action potential properties. RTN neurons in slices from *Scn1a*$^{+/+}$ and *Scn1a*$^{+/-}$ mice (10 days postnatal) were held at a -80 mV and the first action potential elicited by a +120 pA depolarizing current injection were selected for analysis. **A-C**, average (n = min. of 7 action potentials/condition) action potentials (top) and first-time derivatives of action potentials (bottom) recorded from RTN neurons in slices from *Scn1a*$^{+/+}$ and *Scn1a*$^{+/-}$ mice under control conditions (**A**) and in the presence of Hm1a (50 μM) (**B-C**). Right, corresponding phase plots of the voltage and derivative traces show that under these experimental conditions RTN neurons from each genotype show similar action potential properties. **D-F,** Summary data shows *Scn1a*$^{+/+}$ and *Scn1a*$^{+/-}$ mice exhibit similar spike threshold ($p > 0.05$) (**D**), maximum rate of depolarization ($p > 0.05$) (**E**) and spike amplitude ($p > 0.05$) (**F**). Results were compared by one way ANOVA and the Tukeys multiple comparison test.

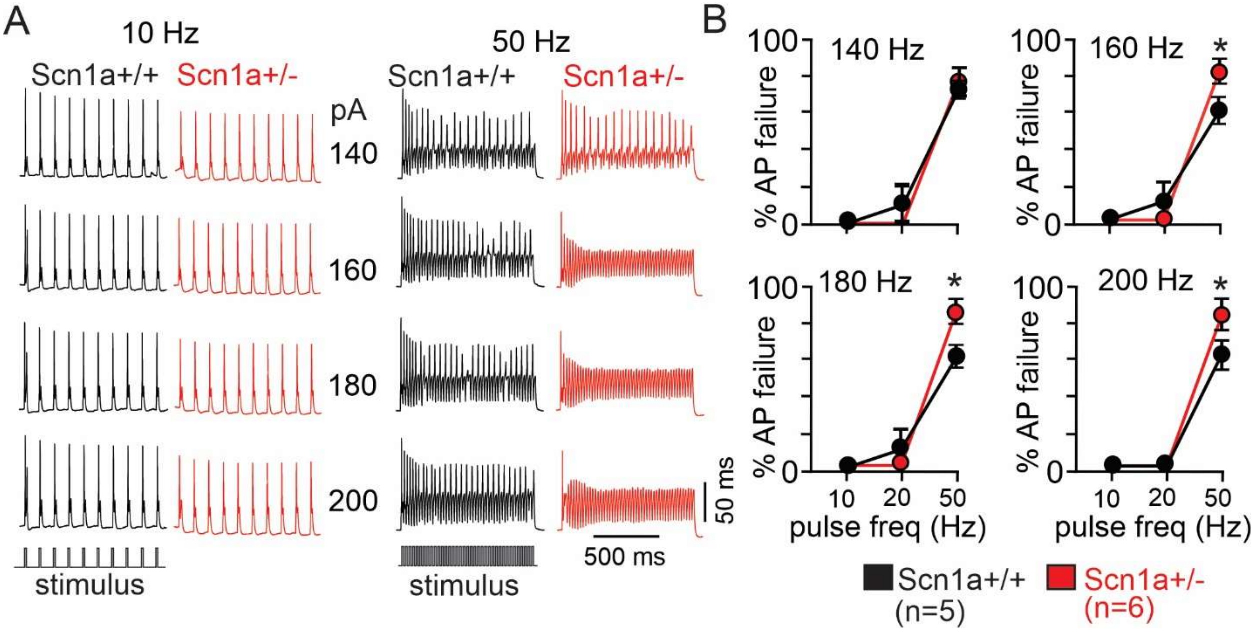

Supplementary Figure 7. RTN neurons in slices from *Scn1a*$^{+/-}$ mice show a limited ability to respond to high frequency stimulation. A, traces of membrane potential recorded in whole-cell current-clamp show the firing response of RTN neurons to 1-second-long trains of depolarizing pulses (140-200 pA) at 10 and 50 Hz. **B**, summary data plotted as percent action potential failure (depolarizing stimulus that do not elicit an action potential). RTN neurons in slices from each genotype show a similar low failure rate when stimulated at between 10-20 Hz. However, RTN neurons in slices from *Scn1a*$^{+/-}$ mice failed more frequently than neurons in control tissue at 50 Hz stimulation. *, $p < 0.05$; means are compared using repeated measures two -way ANOVA followed by Sidak multiple comparison test
